# CFD Analysis to Study Effect of Circular Vortex Generator Placed in Inlet Section to Investigate Heat Transfer Aspects of Solar Air Heater

**DOI:** 10.1155/2014/567257

**Published:** 2014-08-31

**Authors:** Vipin B. Gawande, A. S. Dhoble, D. B. Zodpe

**Affiliations:** Department of Mechanical Engineering, Visvesvaraya National Institute of Technology, Nagpur, India

## Abstract

CFD analysis of 2-dimensional artificially roughened solar air heater duct with additional circular vortex generator, inserted in inlet section is carried out. Circular transverse ribs on the absorber plate are placed as usual. The analysis is done to investigate the effect of inserting additional vortex generator on the heat transfer and flow friction characteristics inside the solar air heater duct. This investigation covers relative roughness pitch in the range of 10 ≤ *P/e* ≤ 25 and relevant Reynolds numbers in the range of 3800 ≤ Re ≤ 18000. Relative roughness height (*e/D*) is kept constant as 0.03 for analysis. The turbulence created due to additional circular vortex generator increases the heat transfer rate and at the same time there is also increase in friction factor values. For combined arrangement of ribs and vortex generator, maximum Nusselt number is found to be 2.05 times that of the smooth duct. The enhancement in Nusselt number with ribs and additional vortex generator is found to be 1.06 times that of duct using ribs alone. The maximum increase in friction factor with ribs and circular vortex generator is found to be 2.91 times that of the smooth duct. Friction factor in a combined arrangement is 1.114 times that in a duct with ribs alone on the absorber plate. The augmentation in Thermal Enhancement Factor (TEF) with vortex generator in inlet section is found to be 1.06 times more than with circular ribs alone on the absorber plate.

## 1. Introduction

A solar air heater uses clean and sustainable solar energy and converts it into usable thermal energy. Solar air heaters are widely used for drying of agricultural, textile, and marine products. They are also used for heating of buildings to maintain a comfortable environment in the winter season. In addition, they are also widely used in devices having applications in chemical, pharmaceuticals, oil industries, and space heating.

Solar air heater is operating on the principle of forced convective heat transfer between the wall and a working fluid (air). But the efficiency of the air heater is naturally of low value due to the fact that air has inferior thermodynamics properties in terms of heat transfer. The method used to increase the heat transfer coefficient between the working fluid (air) and absorber surface is to create the turbulence inside the solar air heater duct. The turbulence is used to break the viscous sublayer at the absorber surface. The turbulence is created by providing surface roughness on the heat transferring surface. Several researchers have conducted many experimental and numerical studies to study the effect of surface roughness on the thermal behavior in solar air heater channel. Saini [1] reported that the height of the roughness element should be kept small in comparison with the duct dimension. This is due to the fact that although the application of artificial roughness results in higher heat transfer enhancement, this arrangement also causes increase in friction losses leading to excessive power requirements for the air flow through the duct.

In the experimental investigations reported in the literature, Prasad and Mullick [2] have carried out experiments using small diameter wires as an artificial roughness. Wires were attached on the flow side of absorber plate for improving thermal behavior in solar air hater duct. Experiment was conducted by Sparrow and Hossfeld [3] to determine the heat transfer, pressure drop, and flow field responses to the rounding of the peaks of a corrugated-wall duct. They observed that the rounding of the corrugation peaks brought about a decrease in the Nusselt number at a given Reynolds number. At the same time friction factor is also found to be decreased corresponding to a given Reynolds number. In another experiment conducted by Verma and Prasad [4], roughness elements in the form of circular wires of different diameters were provided on the absorber plate at varying pitches. They found that the value of heat transfer enhancement factor varies between 1.25 and 2.08 within the range of parameters investigated. Yadav and Kaushal [5] studied the effect of heat transfer and friction characteristics of turbulent flow of air. The air is passed through a rectangular duct having absorber plate with circular protrusion as a roughness, arranged in the form of the angular arc. Experiment was conducted using Reynolds number in the range of 3600 to 18000, *P*/*e* in the range of 12 to 24, *e*/*D* in the range of 0.015 to 0.030, and arc angle in the range of 45° to 75°. They reported maximum enhancement in heat transfer and friction factor as 2.89 and 2.93 times as compared with smooth duct. Several investigators have carried out various experiments using different roughness geometries like ribs, wire mesh, dimple shaped geometry, arc shaped ribs, metal grit ribs, W-shaped ribs, solid, porous and perforated baffles, delta winglet, impinging jets, and so forth. The detailed description regarding these geometries can be obtained in a review paper by Gawande et al. [6].

Apart from this, many researchers have carried out numerical analysis of solar air heater duct using various versions of ANSYS FLUENT. Chaube et al. [7] used FLUENT 6.1 CFD code and SST *k*-*ε* as a turbulence model for analysis of ten different ribs, namely, rectangular, square, chamfered, triangular, and so forth. Higher heat transfer was achieved with chamfered ribs and rectangular ribs of size 3 × 5 give the best performance index for the range of parameters investigated. FLUENT 6.3.26 code and renormalization group (RNG) *k*-*ε* turbulence model were used by Kumar and Saini [8] to simulate artificially roughened solar air heater having an arc shaped artificial roughness. Karmare and Tikekar [9] has carried out simulation on a solar air heater duct with an absorber plate roughened with metal ribs of circular, square, and triangular cross section, having 60° inclinations to the air flow. Their simulation reported that square cross-section ribs with 58° angle of attack gives maximum heat transfer for the range of operating parameters investigated. Solar air heater duct with wedge-shaped transverse ribs roughness was simulated using FLUENT code by Gandhi and Singh [10]. Their study shows that there is a good agreement between the experimental and numerical analysis values. Sharma and Thakur [11] have simulated solar air heater duct with V-shaped ribs roughness on the flow side of the absorber plate. They found that the increase in heat transfer was mainly due to the combined effect of swirling motion, detachment, and attachment of the fluid. Recently Yadav and Bhagoria [12–18] have carried out CFD analysis of various geometries including triangular, circular transverse wire ribs, square sectioned transverse ribs, and equilateral triangular sectioned ribs. Yadav and Bhagoria have simulated the above geometries using ANSYS FLUENT code and RNG *k*-*ε* turbulence model. The results were presented in terms of Nusselt number versus Re, friction factor versus Re, Nusselt number ratio versus Re, friction factor ratio versus Re, and thermal enhancement factor versus Re.

From the literature review cited above, it is revealed that both experimental and numerical analysis works ar e done using the roughness geometries fitted on the flow side of absorber plate. Promvonge et al. [19] have adopted a new concept to increase the heat transfer in solar air heater duct. Promvonge et al. used ribs as artificial roughness on the absorber plate and in addition to this delta-winglet, as a swirl flow generator in inlet section to create the additional turbulence from inlet section. This concept results in increase of heat transfer rate. Adopting the concept given by Promvonge, the present analysis is carried out. The objective of the present work is

1. to investigate the heat transfer and flow friction characteristics in a solar air heater, using circular transverse wire ribs as a roughness underneath the absorber plate and the same circular transverse wire rib as a vortex generator in inlet section of the duct,
2. to investigate the effect of relative roughness pitch (*P*/*e*) on the average Nusselt number, average friction factor, and thermal enhancement factor keeping relative roughness height (*e*/*D* = 0.03) constant,
3. to compare the present results with the results having only circular transverse ribs as a roughness and with the smooth duct.

## 2. CFD Simulation

CFD simulation of two-dimensional artificially roughened solar air heater duct along with circular rib as a vortex generator in inlet section is carried out using ANSYS FLUENT 14.5. The general assumptions considered for the analysis are as follows.

1. The flow is considered as being steady, two-dimensional, and turbulent.
2. The flow is single phase across the duct.
3. The walls, in contact with the fluid, are assigned no-slip boundary condition.
4. The thermodynamics properties of both the air and absorber plate (aluminum) are considered constant.
5. Radiation heat transfer is considered negligible in the analysis.

### 2.1. Computational Solution Domain

**Figure 1:**
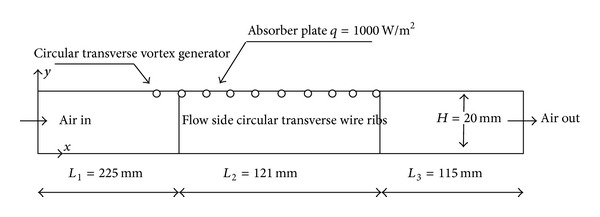
Schematic of two-dimensional solution domain for CFD analysis.

A computational model created in ANSYS workbench 14.5 used for the analysis is shown in Figure 1. The solution domain is created as per ASHRAE standard and is adopted from details suggested in Chaube et al. [7]. Researchers have indicated that the results obtained from most of the cases of two-dimensional model are in good agreement with those of the experimental investigations. Apart from this, two-dimensional analysis requires less computational time and memory. In view of this, it is decided to carry out the present analysis using two-dimensional model.

**Figure 2:**
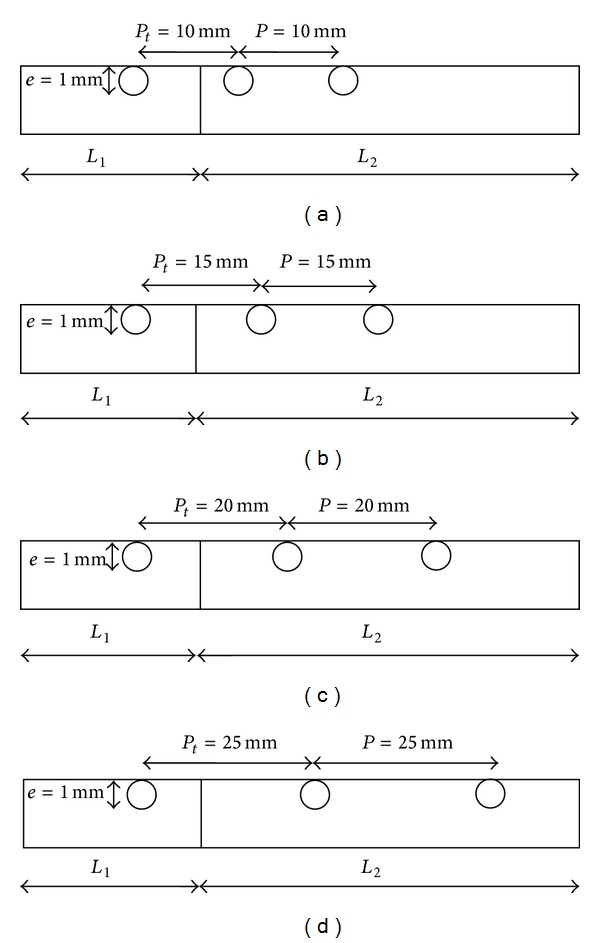
Roughened absorber plate with circular transverse ribs at a pitch of (a) *P* = 10 mm, (b) *P* = 15 mm, (c) *P* = 20 mm, (d) *P* = 25 mm, and circular vortex generator in inlet section at a transverse pitch of (a) *P*_*t*_ = 10 mm, (b) *P*_*t*_ = 15 mm, (c) *P*_*t*_ = 20 mm, and (d) *P*_*t*_ = 25 mm.

As per the ASHRAE guidelines, the solar air heater duct is divided into three sections, namely, inlet (*L*_1_), test (*L*_2_), and exit (*L*_3_) sections. The internal cross section of the duct is 100 × 20 mm^2^. Circular transverse rib and circular vortex generator in inlet section is having rib height (*e*) of 1 mm. The pitch (*P*) for circular transverse ribs is varied from 10 mm to 25 mm in four values. The circular vortex generator fitted in inlet section is kept at transverse pitch (*P*_*t*_) of 10 mm, 15 mm, 20 mm, and 25 mm as shown in Figure 2. The relative roughness height (*e*/*D*) is kept constant at 0.03 and relative roughness pitch (*P*/*e*) varies from 10 to 25. The solar air heater shows better thermohydraulic performance in the Reynolds number range of 3800–18000, as reported by Kumar and Saini [8]. Hence, the same specified range of Reynolds number is adopted for the present analysis. The geometrical and operating parameters used in the CFD analysis of the duct are listed in Tables 1 and 2, respectively.

### 2.2. Grid Generation and Validation of the Model

**Figure 3:**
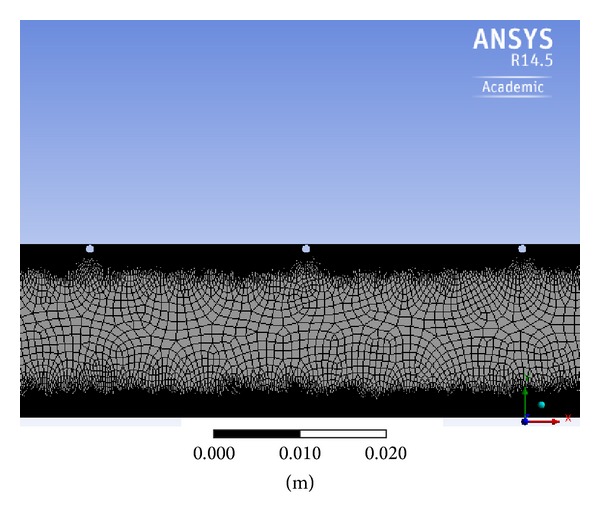
Two-dimensional nonuniform grid.

The mesh employed for numerical simulation plays an important role towards determining the accuracy of the predicted solution. Nonuniform computational quad grid structure generated in ANSYS 14.5 is used here for the numerical solutions. The grid is made fine near the walls and coarser in the middle of the geometry to capture the effect of boundary layer. A grid of 156453 cells is adopted for the analysis after a careful check of Nusselt number and friction factor values. Nonuniform grid for solar air heater duct is shown in Figure 3.

**Figure 4:**
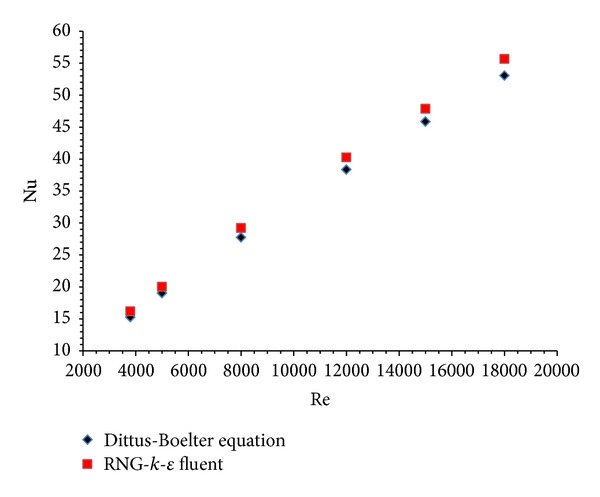
Validation of Dittus-Boelter empirical correlation with RNG-*k*-*ε* FLUENT model.

**Figure 5:**
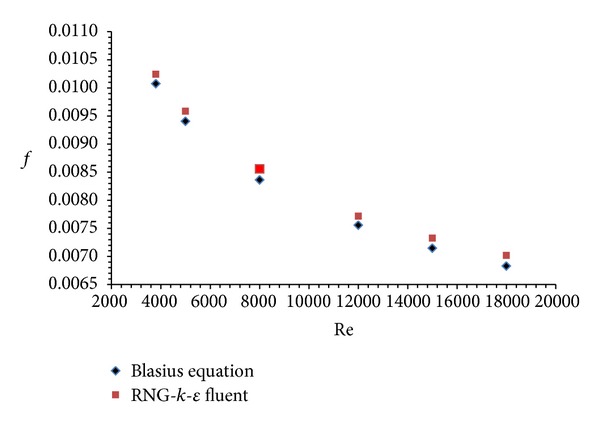
Validation of Blasius empirical correlation with RNG-*k*-*ε* FLUENT model.

FLUENT code is used to test smooth duct using RNG-*k*-*ε* turbulence model without considering any artificial roughness on the absorber plate. The results thus obtained are compared with the Dittus-Boelter empirical correlation for Nusselt number and with Blasius empirical correlation for friction factor. The comparison between Nusselt number and friction factor values with empirical correlations for smooth duct is shown in Figures 4 and 5, respectively. The comparison shows a good agreement between results obtained from FLUENT and values of empirical correlations. This ensures the correctness of the numerical data obtained from the present work. Similar type of trend in results was observed by Yadav and Bhagoria [13]. This allows the validation of results and suggests that further analysis can be carried out for more detailed investigations.

### 2.3. Governing Fluid Flow Equations and Data Reduction

The governing equations of continuity, conservation of momentum, and energy are used to solve the forced turbulent fluid flow and heat transfer in the artificially roughened solar air heater duct. The governing equations in rectangular Cartesian coordinate system are well known and can be written as follows, considering flow to be two-dimensional, steady with incompressible fluid, and further negligible radiation heat transfer from the duct to the surrounding.

Continuity equation is as follows:

(1) $$\begin{array}{c} \frac{\partial u}{\partial x}+\frac{\partial v}{\partial y}=0. \end{array}$$

Momentum equation is as follows:

(2) $$\begin{array}{c} u\frac{\partial u}{\partial x}+v\frac{\partial u}{\partial y}=-\frac{1}{\rho }\frac{\partial p}{\partial x}+\upsilon \left(\frac{\partial^{2}u}{\partial x^{2}}+\frac{\partial^{2}u}{\partial y^{2}}\right),\\ u\frac{\partial v}{\partial x}+v\frac{\partial v}{\partial y}=-\frac{1}{\rho }\frac{\partial p}{\partial y}+\upsilon \left(\frac{\partial^{2}v}{\partial x^{2}}+\frac{\partial^{2}v}{\partial y^{2}}\right). \end{array}$$

Energy equation is as follows:

(3) $$\begin{array}{c} u\frac{\partial T}{\partial x}+v\frac{\partial T}{\partial y}=\alpha \left(\frac{\partial^{2}T}{\partial x^{2}}+\frac{\partial^{2}T}{\partial y^{2}}\right), \end{array}$$

where *υ* is the kinematic viscosity and *α* is the thermal diffusivity.

Use of circular transverse ribs along with circular vortex generator results in enhancement in heat transfer which is predicted by calculating average Nusselt number. This enhancement is also accompanied by an increase in friction factor. The average Nusselt number for artificially roughened solar air heater is computed as

(4) $$\begin{array}{c} {\text{Nu}}_{r}=\frac{hD}{k}, \end{array}$$

where *h* is convective heat transfer coefficient.

The average friction factor for artificially roughened solar air heater is computed by

(5) $$\begin{array}{c} f_{r}=\frac{\left(\Delta P/l\right)D}{2\rho vU^{2}}, \end{array}$$

where Δ*P* is the pressure drop across the test section length (*l*) of an artificially roughened solar air heater.

The Reynolds number is defined as

(6) Re=ρUDμ.

Thermal enhancement factor (TEF) proposed by Webb and Eckert [20] is used to evaluate the enhancement in heat transfer of a roughened solar air heater duct compared to that of the smooth duct for the same pumping power requirement and is given as

(7) $$\begin{array}{c} \text{TEF}=\;\frac{{\text{Nu}}_{r}/{\text{Nu}}_{s}}{{\left(f_{r}/f_{s}\right)}^{1/3}}. \end{array}$$

Nusselt number for smooth duct (Nu_*s*_) of a solar air heater can be obtained by the Dittus-Bolter equation [21],

(8) $$\begin{array}{c} {\text{Nu}}_{s}=0.023\text{R}{\text{e}}^{0.8}\text{P}{\text{r}}^{0.4}. \end{array}$$

Friction factor for smooth duct (*f*_*s*_) of a solar air heater can be obtained by Blasius equation [22],

(9) $$\begin{array}{c} f_{s}=0.0791\text{R}{\text{e}}^{-0.25}. \end{array}$$

### 2.4. Boundary Conditions and Selection of Turbulence Model

Two-Dimensional computational domain of roughened solar air heater duct is divided into inlet, outlet, and wall boundaries. Air is used as a working fluid. The material of the absorber plate is taken as aluminum. Both air and absorber plates are assumed to remain at constant average bulk temperature. Thermophysical properties are given in Table 3 for working fluid air and absorber plate material (aluminum).

At the inlet of the solution domain, velocity inlet boundary condition is specified in FLUENT. The upper wall in the test section is considered as an absorber plate and assigned a heat flux of 1000 W/m^2^. The other walls are assigned no-slip boundary condition and are adiabatic. The circular transverse ribs on the absorber plate and circular vortex generator in inlet section are also considered adiabatic. The working fluid (Air) enters at a temperature of 300 K in the beginning. At the outlet, pressure outlet boundary condition is applied with a value of 1.013 × 10^5^ N/m^2^. In FLUENT, RNG-*k*-*ε* turbulence model is taken for analysis since it gives results very close to the experimental results as reported by researchers [12, 13]. Discretization of the governing equation is done with SIMPLE (semi-implicit method for pressure linked equations) algorithm given by Patankar [23]. A Second-order upwind scheme is used for all the transport equations as suggested by Karmare and Tikekar [9]. For continuity equation, a convergence criterion of 10^−3^ is assigned and a convergence criterion of 10^−6^ is assigned for velocity components and energy.

## 3. Results and Discussion

CFD analysis of roughened solar air heater duct using circular transverse ribs as roughness on flow side of absorber plate and circular vortex generator in inlet section is conducted. It is observed that this combination causes the following two effects.

1. Recirculating flow is induced by the ribs on the absorber plate.
2. Vortex flow is created by the circular vortex generator in inlet section.

The additional turbulence created by the circular transverse vortex turbulator in inlet section is found to be very effective in the vicinity of the absorber plate. The use of these turbulators leads to better mixing of fluid between the heated wall surface and the core and thereby enhances the heat transfer rate in the solar air heater duct.

### 3.1. Effect of Combined Circular Transverse Ribs and Circular Vortex Generator

#### 3.1.1. Heat Transfer

**Figure 6:**
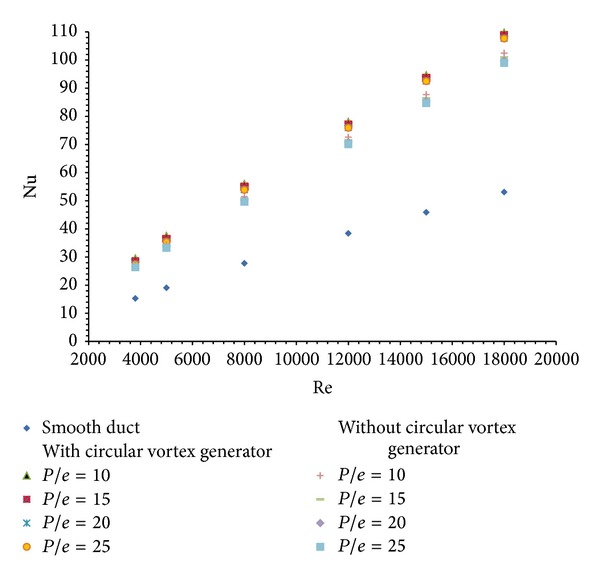
Variation of average Nusselt number (Nu) with Reynolds number (Re).

The effect of relative roughness pitch (*P*/*e*) using circular transverse ribs alone on the absorber plate is studied in terms of average Nusselt number, average friction factor, and thermal enhancement factor.  Figure 6 shows the enhancement in heat transfer in terms of average Nusselt number values at constant value of relative roughness height (*e*/*D* = 0.03) when circular vortex generator is not inserted in inlet section of duct. It is found that Nu value increases with the rise of Re. The turbulence is created by the ribs which in turn break the laminar sublayer thickness and reduces thermal resistance in the direction of flow. This decrease in thermal resistance yields the increase in Nu with the increase in the Reynolds number. The maximum enhancement in heat transfer is found for *P*/*e* = 10 and then Nu decreases with the increase in pitch values. Maximum enhancement in Nusselt number is found to be 1.92 times that of the smooth duct corresponding to *P*/*e* = 10 and Reynolds number of 18,000. The results are very much close to the results previously obtained by Yadav and Bhagoria [13].

The insertion of circular vortex generator helps to produce additional turbulence in the flow field which further increases the mixing between the cold fluid in the core of the duct and the hot fluid near the absorber plate. This in turn helps in increasing heat transfer rate.  Figure 6 also shows the variation of average Nusselt number using combined ribs and vortex generator in inlet section. It is found that there is increase in average Nusselt number for the increase in Reynolds number. In this combination of ribs and vortex generator, maximum Nusselt number is found to be 2.05 times that of the smooth duct. The enhancement in average Nusselt number with ribs with vortex generator is found to be 1.06 times that of duct using ribs alone.

**Figure 7:**
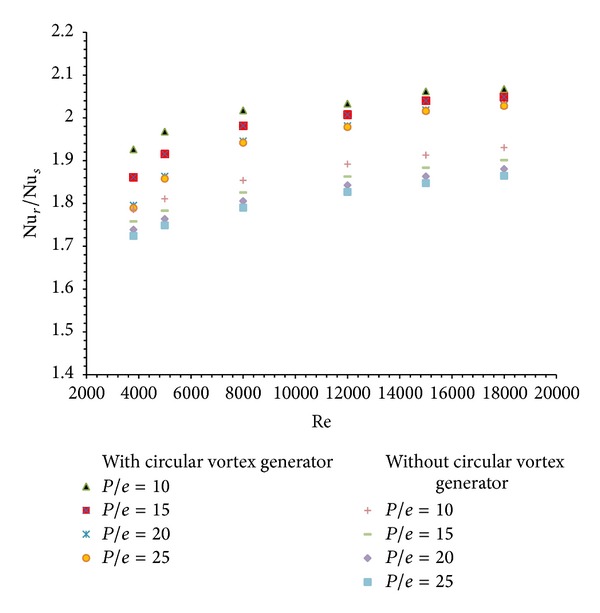
Variation of average Nusselt number enhancement ratio (Nu_*r*_/Nu_*s*_) with Reynolds number (Re).

Nusselt number enhancement ratio (Nu_*r*_/Nu_*s*_) is the ratio of Nusselt number for duct with roughened absorber plate to the Nusselt number for smooth duct. The variation of average Nusselt number enhancement ratio for air heater duct with ribs alone is shown in Figure 7. It is observed that the average Nusselt number enhancement ratio increases with increase in Reynolds number. It is maximum for *P*/*e* = 10 and its value is (Nu_*r*_/Nu_*s*_) = 1.9307 for the Reynolds number of 18000.

Similar trend in increase of average Nusselt number enhancement ratio is found in case of air heater duct with combined rib and vortex generator fitted in inlet section. It is also shown in Figure 7. Here again the maximum enhancement in average Nusselt number ratio is obtained for *P*/*e* =10 and for Reynolds number of 18000. Its value is found to be (Nu_*r*_/Nu_*s*_) = 2.067. So there is a 1.07 times increase in enhancement is observed using a single row of circular vortex generator in inlet section along with circular transverse ribs on the absorber plate.

**Figure 8:**
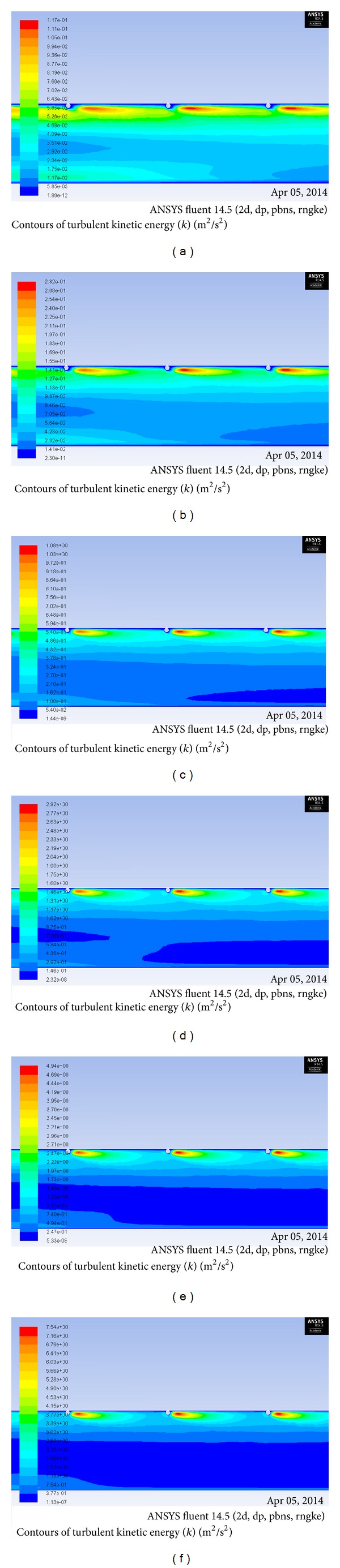
Contour plot of turbulence kinetic energy for *P*/*e* = 25, *e*/*D* =0.03 for Reynolds number of (a) Re = 3800, (b) Re = 5000, (c) Re = 8000, (d) Re = 12000, (e) Re =15000, and (f) Re = 18000.

**Figure 9:**
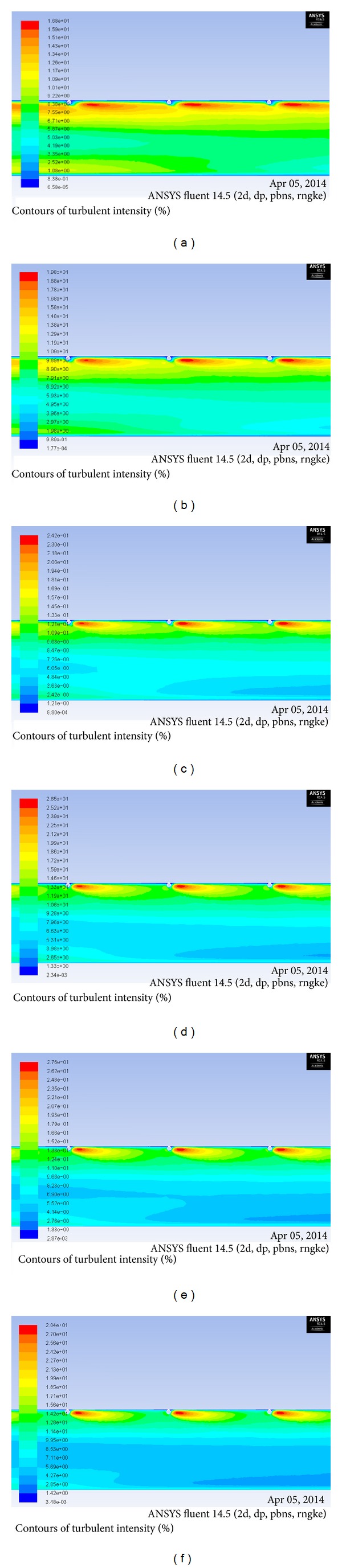
Contour plot of turbulence intensity for *P*/*e* = 25, *e*/*D* = 0.03 for Reynolds number of (a) Re = 3800, (b) Re = 5000, (c) Re = 8000, (d) Re = 12000, (e) Re =15000, and (f) Re = 18000.

Turbulent kinetic energy increases with increase in Reynolds number. This means that increased turbulent dissipation rate causes increase in turbulence intensity. This in turn results in increase of Nusselt number. Turbulence kinetic energy contour plots can be used to understand the heat transfer phenomenon in a better way. Turbulent kinetic energy contour plot for *P*/*e* = 25, *e*/*D* = 0.03 and for different range of Reynolds number is shown in Figure 8. The maximum value of turbulent kinetic energy is obtained near the top heated wall on the downstream side of the rib. The intensity of turbulent kinetic energy decreases as the distance from the wall increases. From the figure, it is clear that the turbulent kinetic energy increases as the pitch between the ribs increases. Since at high Reynolds number the turbulent kinetic energy is higher, which reduces thermal resistance in the flow by breaking laminar sublayer, Nusselt number is found to be maximum at Re = 18000 and *P*/*e* = 25. Similarly, the turbulent intensity increases with increase with Reynolds number leading to high turbulence generation inside the duct. This turbulence causes mixing of core and fluid from heated absorber plate which leads to the enhancement of heat transfer in the duct. The turbulent intensity is also found to increase near the top heated wall on the downstream side of the rib and it decreases as the distance from the wall increases. The contours plot of turbulent intensity for *P*/*e* = 25, *e*/*D* = 0.03 for different values of Reynolds numbers is shown in Figure 9. From the figure it is concluded that the turbulent intensity increases with the increase in Reynolds number and is higher for Re = 18000 for the range of parameters investigated for the present analysis.

#### 3.1.2. Friction Factor

**Figure 10:**
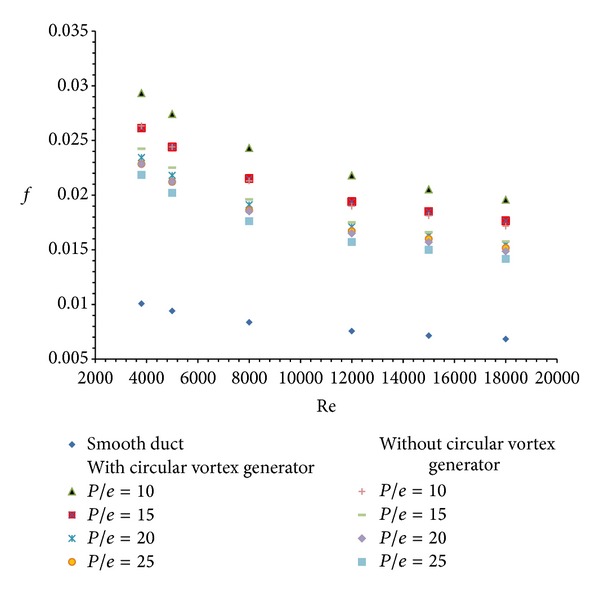
Variation of average friction factor (*f*) with Reynolds number (Re).

The provision of circular transverse ribs increases the intensity of turbulence that breaks the laminar sublayer. This suppression of viscous sublayer causes decrease in friction factor values as the Reynolds number increases. Friction factor values for roughened absorber plate are higher as compared to those of the smooth solar air heater. The higher intensity of turbulence generated with the increase in Reynolds number in the duct decreases friction factor. The variation of average friction factor with Reynolds number for different values of varying relative roughness pitch (*P*/*e*) using ribs alone is shown in Figure 10. From this figure, it is concluded that the maximum enhancement in average friction factor values is for *P*/*e* = 10 and Reynolds number of 3800. The enhancement in friction factor is found to be 2.61 times that of the smooth duct.

Similar decrease in average friction factor values is also observed in case of solar heater duct equipped with ribs and vortex generator in inlet section. The variation of average friction factor with Reynolds number for this particular case is also plotted in Figure 10. Here also the maximum friction factor is obtained at *P*/*e* =10 and Reynolds number of 3800. The enhancement here is found to be 2.91 times that of the smooth duct.

**Figure 11:**
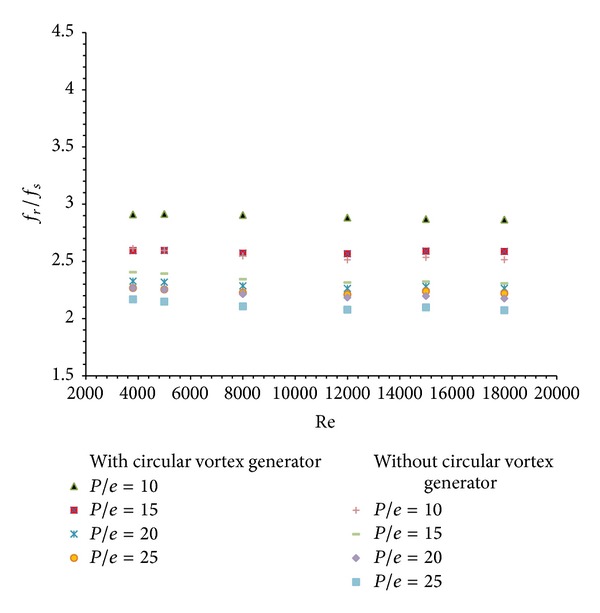
Variation of average friction factor enhancement ratio (*f*_*r*_/*f*_*s*_) with Reynolds Number (Re).

Friction factor enhancement ratio (*f*_*r*_/*f*_*s*_) is the ratio of friction factor for roughened solar air heater duct to the friction factor for smooth solar air heater duct. In case of solar air heater duct with circular transverse ribs alone, the average friction factor enhancement ratio is found to be maximum as 2.61 for *P*/*e* = 10 and at Reynolds number of 3800. This is shown in Figure 11. Similar results are achieved for the solar air heater duct with combined rib and vortex generator. Here the maximum average friction factor enhancement ratio is obtained at Re = 3800 as 2.91 for *P*/*e* = 10. The variation of average friction factor enhancement ratio for this situation is also shown in Figure 11.

#### 3.1.3. Thermal Enhancement Factor

**Figure 12:**
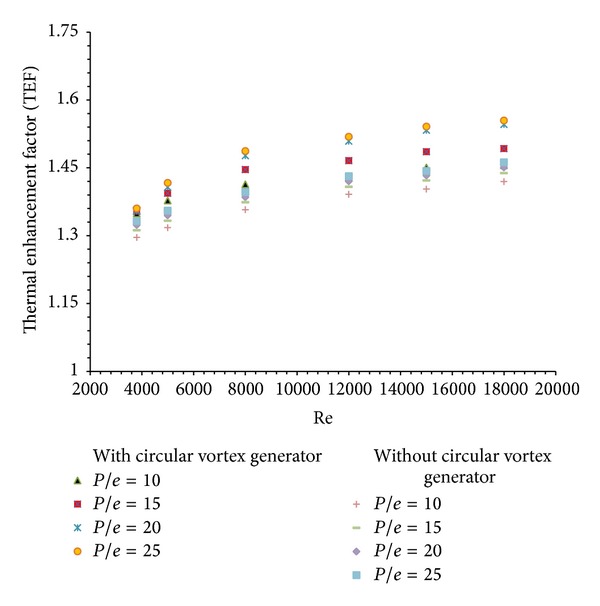
Variation of thermal enhancement factor (TEF) with Reynolds number (Re).

Thermal enhancement factor (TEF) is a measure of predicting the best optimum value for solar air heater in terms of heat transfer and friction factor. It helps in selecting optimal rib dimension and arrangement that will correspond to maximum enhancement in heat transfer with minimum friction power penalty. The variation of thermal enhancement factor with Reynolds number considering the circular transverse rib on absorber plate alone is shown in Figure 12. Similar pattern of increase in TEF with Reynolds number is seen in case of arrangement of ribs along with circular vortex generator in inlet section. It is also plotted in Figure 12. The use of circular vortex generator leads to increase in heat transfer rate with minimum penalty of friction factor. The enhancement with vortex generator in inlet section is found to be 1.06 times more than that with circular ribs alone on the absorber plate.

## 4. Conclusion

A two-dimensional solar air heater duct having constant relative roughness height (*e*/*D*) of 0.03 is analyzed for different values of relative roughness pitch (*P*/*e*), Reynolds number (Re), and inserting circular vortex generator in the inlet section in addition to circular transverse ribs on the flow side of the absorber plate. The following conclusions are drawn from the analysis carried out in this paper.

1. Turbulence plays a vital role in the heat transfer enhancement. The same concept is adopted and an additional circular vortex generator is inserted in inlet section to create additional turbulence in solar air heater duct.
2. The turbulence created by the circular vortex generator causes increase in heat transfer enhancement and at the same time results in increase in pumping power.
3. For combined arrangement of ribs and vortex generator, maximum Nusselt number is found to be 2.05 times that of the smooth duct. The enhancement in Nusselt number with ribs and vortex generator is found to be 1.06 times that of duct using ribs alone.
4. The enhancement in friction factor in a duct with ribs alone is found to be 2.61 times that of the smooth duct, whereas the enhancement with ribs and circular vortex generator is found to be 2.91 times that of the smooth duct. Friction factor in a combined arrangement is 1.114 times that in a duct with ribs alone on the absorber plate.
5. Maximum friction factor enhancement ratio of 2.91 is obtained with combined arrangement at Re = 3800 and *P*/*e* = 10.
6. The enhancement in thermal enhancement factor (TEF) with vortex generator in inlet section is found to be is 1.06 times more than with circular ribs alone on the absorber plate.

## Figures and Tables

**Table 1:** Geometrical parameters for both geometries used in artificially roughened solar air heater duct.

Geometrical parameters	Value
Inlet length of duct (*L*_1_)	225 mm
Test length of duct (*L*_2_)	121 mm
Outlet length of duct (*L*_3_)	115 mm
Width of duct (*W*)	100 mm
Height of duct (*H*)	20 mm
Hydraulic diameter (*D*) of duct	33.33 mm
Rib height (*e*)	1 mm
Pitch (*P*)	10, 15, 20, and 25 mm
Transverse pitch (*P*_*t*_)	10, 15, 20, and 25 mm

**Table 2:** Range of operating parameters for both geometries used in artificially roughened solar air heater duct.

Operating parameters	Range
Uniform heat flux, “*q*”	1000 W/m^2^
Reynolds number, “Re”	3800–18000 (6 values)
Prandtl number, “Pr”	0.7
Relative roughness pitch, “*P*/*e*”	10–25 (4 values)
Relative roughness height “*e*/*D*”	0.03
Duct aspect ratio “*W*/*H*”	5

**Table 3:** Thermophysical properties of air and absorber plate for CFD analysis.

Properties	Air	Absorber plate (aluminum)
Specific heat “*C*_*p*_” (J kg^−1^ K^−1^)	1006.43	871
Thermal conductivity “*k*” (Wm^−1^K^−1^)	0.0242	202.4
Density “*ρ*” (kg m^−3^)	1.225	2719
Viscosity “*μ*” (N m^−2^)	1.7894*e* ^−05^	—

## Nomenclature

*D*: Equivalent or hydraulic diameter of duct, mm
*e*: Rib height, mm
*h*: Heat transfer coefficient, W/m^2^K
*H*: Depth of the duct, mm
*k*: Thermal conductivity of air, W/mK
*L*_1_: Inlet length of duct, mm
*L*_2_: Test length of the duct, mm
*L*_3_: Outlet length of duct, mm
*P*: Pitch, mm
*P*_*t*_: Transverse pitch for vortex generator, mm
*T*: Air temperature, *K*
*U*: Mean airflow velocity in the duct, m/s
*u*: Air flow velocity in *x*-direction, m/s
*v*: Air flow velocity in *y*-direction, m/s
*W*: Width of the duct, mm
Δ*P*: Pressure drop, Pa
*C*_*p*_: Specific heat of air, J/kgK.

### Dimensionless Parameters

*e*/*D*: Relative roughness height
*f*: Friction factor
*f*_*r*_: Friction factor for rough surface
*f*_*s*_: Friction factor for smooth surface
Nu: Nusselt number
Nu_*r*_: Nusselt number for rough surface
Nu_*s*_: Nusselt number for smooth surface
*Pr*⁡: Prandtl number
*P*/*e*: Relative roughness pitch
*Re*: Reynolds number
*W*/*H*: Duct aspect ratio.

### Greek Symbols

*μ*: Dynamic viscosity, Ns/m^2^
*ρ*: Density of air, Kg/m^3^
*k*: Turbulent kinetic energy, m^2^/s^2^
*α*: Thermal diffusivity
*υ*: Kinematic viscosity, m^2^/s.

### Subscripts

*r*: Roughened
*s*: Smooth
CFD: Computational fluid dynamics
TEF: thermal enhancement factor.

## References

[1] Saini JS Use of artificial roughness for enhancing performance of solar air heater.

[2] Prasad K, Mullick SC (1983). Heat transfer characteristics of a solar air heater used for drying purposes. *Applied Energy*.

[3] Sparrow EM, Hossfeld LM (1989). Effect of rounding of protruding edges on heat transfer and pressure drop in a duct. *International Journal of Heat and Mass Transfer*.

[4] Verma SK, Prasad BN (2000). Investigation for the optimal thermohydraulic performance of artificially roughened solar air heaters. *Renewable Energy*.

[5] Yadav S, Kaushal M (2013). Nusselt number and friction factor correlations for solar air heater duct having protrusions as roughness elements on absorber plate. *Experimental Thermal and Fluid Science*.

[6] Gawande VB, Dhoble AS, Zodpe DB (2014). Effect of roughness geometries on heat transfer enhancement in solar thermal systems—a review. *Renewable and Sustainable Energy Reviews*.

[7] Chaube A, Sahoo PK, Solanki SC (2006). Analysis of heat transfer augmentation and flow characteristics due to rib roughness over absorber plate of a solar air heater. *Renewable Energy*.

[8] Kumar S, Saini RP (2009). CFD based performance analysis of a solar air heater duct provided with artificial roughness. *Renewable Energy*.

[9] Karmare SV, Tikekar AN (2010). Analysis of fluid flow and heat transfer in a rib grit roughened surface solar air heater using CFD. *Solar Energy*.

[10] Gandhi BK, Singh KM (2010). Experimental and numerical investigations on flow through wedge shape rib roughened duct. *Journal of the Institution of Engineers (India) Journal*.

[11] Sharma AK, Thakur NS (2012). CFD based fluid flow and heat transfer analysis of a v- shaped roughened surface solar air heater. *International Journal of Engineering Science and Technology*.

[12] Yadav AS, Bhagoria JL (2013). A CFD analysis of a solar air heater having triangular rib roughness on the absorber plate. *International Journal of ChemTech Research*.

[13] Yadav AS, Bhagoria JL (2013). A CFD (computational fluid dynamics) based heat transfer and fluid flow analysis of a solar air heater provided with circular transverse wire rib roughness on the absorber plate. *Energy*.

[14] Yadav AS, Bhagoria JL (2013). A CFD based heat transfer and fluid flow analysis of a conventional solar air heater. *Journal of Engineering Science and Management Education*.

[15] Yadav AS, Bhagoria JL (2013). Numerical investigation of flow through an artificially roughened solar air heater. *International Journal of Ambient Energy*.

[16] Yadav AS, Bhagoria JL (2013). Modeling and simulation of turbulent flows through a solar air heater having square-sectioned transverse rib roughness on the absorber plate. *The Scientific World Journal*.

[17] Yadav AS, Bhagoria JL (2014). A CFD based thermo-hydraulic performance analysis of an artificially roughened solar air heater having equilateral triangular sectioned rib roughness on the absorber plate. *International Journal of Heat and Mass Transfer*.

[18] Yadav AS, Bhagoria JL (2013). Heat transfer and fluid flow analysis of solar air heater: a review of CFD approach. *Renewable & Sustainable Energy Reviews*.

[19] Promvonge P, Khanoknaiyakarn C, Kwankaomeng S, Thianpong C (2011). Thermal behavior in solar air heater channel fitted with combined rib and delta-winglet. *International Communications in Heat and Mass Transfer*.

[20] Webb RL, Eckert ERG (1972). Application of rough surfaces to heat exchanger design. *International Journal of Heat and Mass Transfer*.

[21] McAdams WH (1942). *Heat Transmission*.

[22] Fox W, Pritchard P, McDonald A (2010). *Introduction to Fluid Mechanics*.

[23] Patankar SV (1980). *Numerical Heat Transfer and Fluid Flow*.

